# Rhenium Diselenide (ReSe_2_) Near‐Infrared Photodetector: Performance Enhancement by Selective p‐Doping Technique

**DOI:** 10.1002/advs.201901255

**Published:** 2019-08-27

**Authors:** Jinok Kim, Keun Heo, Dong‐Ho Kang, Changhwan Shin, Sungjoo Lee, Hyun‐Yong Yu, Jin‐Hong Park

**Affiliations:** ^1^ Department of Electrical and Computer Engineering Sungkyunkwan University Suwon 16419 Korea; ^2^ School of Electrical and Electronic Engineering Nanyang Technological University 50 Nanyang Avenue 639798 Singapore Singapore; ^3^ SKKU Advanced Institute of Nano Technology (SAINT) Sungkyunkwan University Suwon 16419 Korea; ^4^ School of Electrical Engineering Korea University Seoul 02841 Korea

**Keywords:** HCl doping, p‐doping, photodetector, ReSe_2_, selective doping, transition‐metal dichalcogenides (TMDs), transistor

## Abstract

In this study, a near‐infrared photodetector featuring a high photoresponsivity and a short photoresponse time is demonstrated, which is fabricated on rhenium diselenide (ReSe_2_) with a relatively narrow bandgap (0.9–1.0 eV) compared to conventional transition‐metal dichalcogenides (TMDs). The excellent photo and temporal responses, which generally show a trade‐off relation, are achieved simultaneously by applying a p‐doping technique based on hydrochloric acid (HCl) to a selected ReSe_2_ region. Because the p‐doping of ReSe_2_ originates from the charge transfer from un‐ionized Cl molecules in the HCl to the ReSe_2_ surface, by adjusting the concentration of the HCl solution from 0.1 to 10 m, the doping concentration of the ReSe_2_ is controlled between 3.64 × 10^10^ and 3.61 × 10^11^ cm^−2^. Especially, the application of the selective HCl doping technique to the ReSe_2_ photodetector increases the photoresponsivity from 79.99 to 1.93 × 10^3^ A W^−1^, and it also enhances the rise and decay times from 10.5 to 1.4 ms and from 291 to 3.1 ms, respectively, compared with the undoped ReSe_2_ device. The proposed selective p‐doping technique and its fundamental analysis will provide a scientific foundation for implementing high‐performance TMD‐based electronic and optoelectronic devices.

## Graphical abstract

**A rhenium diselenide (ReSe_2_) photodetector featuring long wavelength detection, short response time, and high photoresponsivity** is demonstrated by applying a selective HCl p‐doping technique to the ReSe_2_ channel region. The effects of the HCl p‐doping on the ReSe_2_ material and device are thoroughly investigated via Raman spectroscopy, Kelvin probe force microscopy, and electrical measurements in dark and under light illumination conditions.

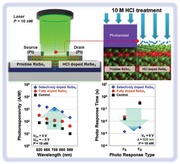

## 1 Introduction

In the past decade, owing to the superior optical properties of transition‐metal dichalcogenides (TMDs), such as their excellent light–matter interaction,^1^ high light absorbance,^2^ and efficient photocarrier generation,^3^, ^4^, ^5^ various TMD materials have been proposed and researched for high‐performance photodetectors. In the initial research stage, group VI atom‐based TMD materials (e.g., MoS_2_, MoSe_2_, WS_2_, and WSe_2_) and a back‐gate transistor structure were suggested for the fabrication of photodetectors.^6^, ^7^, ^8^, ^9^, ^10^, ^11^ These TMD photodetectors exhibited excellent photoresponsivity values between 10^−1^ and 10^4^ A W^−1^, as well as low dark currents between 10^−9^ and 10^−12^ A µm^−1^. Lopez‐Sanchez et al.^6^ and Choi et al.^9^ demonstrated MoS_2_ photodetectors with high photoresponsivity (880 A W^−1^ under 550 nm and 0.12 A W^−1^ under 633 nm) and low dark currents (2 pA^6^ at *V*_G_ = −70 V and 10 pA^9^ at *V*_G_ = −1.2 V). However, these photodetectors based on group VI TMDs were unable to detect infrared light (λ > 900 nm) owing to their wide energy bandgap of >1.2 eV.^12^, ^13^, ^14^ Under this technical circumstance, group VII atom‐based rhenium diselenide (ReSe_2_), which has a relatively narrow bandgap (0.9–1.0 eV),^15^ was suggested for the carrier‐transport region of photodetectors; however, the fabricated ReSe_2_ devices exhibited poor photoresponsivity compared with the group VI TMD photodetectors. The maximum photoresponsivity values of O_2_ plasma‐treated and Mo‐doped ReSe_2_ photodetectors were 95^15^ and 55.5 A W^−1^,^16^ respectively, under 633 nm laser illumination. Recently, by applying (3‐aminopropyl)trimethoxysilane (APTMS)^17^ and triphenylphosphine (PPh_3_)^18^ n‐doping techniques to ReSe_2_ photodetectors, we significantly improved their photoresponsivity to 4.2 × 10^4^ and 1.18 × 10^6^ A W^−1^, respectively. However, the photoresponse time of these n‐doped devices was significantly degraded owing to the reduced depletion width at the metal/ReSe_2_ junction and the consequent reduction in photocarrier collection (τ: 2.7 ms → 4.5 ms after APTMS doping and τ: 64 ms → 263 ms after PPh_3_ doping).

Here, we demonstrated a ReSe_2_ photodetector featuring long wavelength detection, short response time, and high photoresponsivity by applying a p‐doping process based on hydrochloric acid (HCl) to a selected ReSe_2_ region. The built‐in potential at the p^+^/p junction formed by the HCl p‐doping allowed the ReSe_2_ photodetector to have a high photoresponsivity and a short photoresponse time simultaneously. The effects of the HCl p‐doping on the ReSe_2_ material and device were thoroughly investigated via Raman spectroscopy, Kelvin probe force microscopy (KPFM), and electrical measurements in the dark and under light illumination.

## 2 Results and Discussion

**Figure 1:**
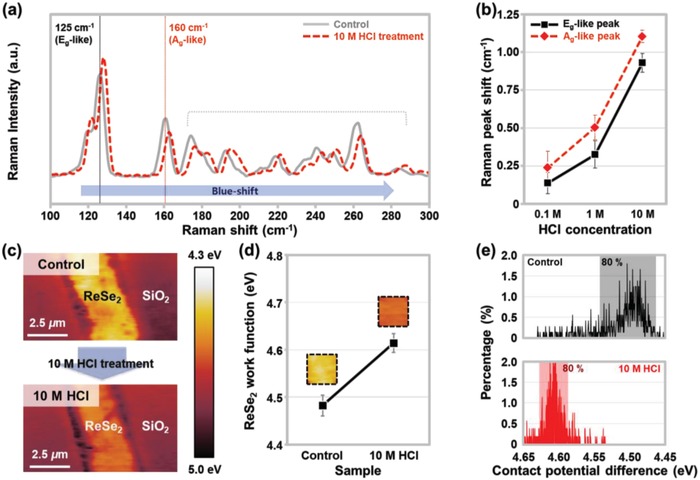
a) Raman spectra of the control and 10 m HCl‐treated ReSe_2_. b) Shifts of the E_g_‐like and A_g_‐like peaks after treatment with HCl of various concentrations (0.1, 1, and 10 m). c) KPFM mapping images, d) work‐function values, and e) contact potential difference (*V*_CPD_) histograms, which were obtained on the surfaces of ReSe_2_ before and after 10 m HCl treatment.

First, we investigated the effect of HCl treatment on the ReSe_2_ crystal material through microscopic analyses, such as Raman spectroscopy and KPFM. **Figure** **1**a shows the Raman spectra measured on the ReSe_2_ material before and after surface treatment with 10 m HCl. Several peaks were observed in the spectral range of 100–300 cm^−1^. This is because the distorted triclinic structure of ReSe_2_ caused 18 Raman‐active vibration modes,^19^ unlike group IV TMDs with an isotropic hexagonal structure (e.g., MoS_2_, WSe_2_, MoSe_2_, and WS_2_).^20^, ^21^, ^22^, ^23^ When 10 m HCl was applied to the ReSe_2_, all the Raman peaks were slightly blue‐shifted (gray solid line → red dotted line). This is likely because the in‐ and out‐of‐plane vibrations of the ReSe_2_ structure were intensified by the HCl treatment.^24^ The blue‐shift phenomenon of the Raman peaks is commonly observed when p‐type dopants donate holes to TMD materials.^25^, ^26^, ^27^ In a previous study, we confirmed that the p‐type doping of WSe_2_ by octadecyltrichlorosilane (OTS) caused the (E^1^_2g_ + A_2g_) peak to blue‐shift by −2.03 cm^−1^.^25^ Furthermore, we found that the peak shift due to the HCl p‐doping was strongly dependent on the concentration of the HCl solution. Figure 1b shows the shifts of the E_g_‐like and A_g_‐like peaks of ReSe_2_ after doping with various HCl concentrations (0.1, 1, and 10 m). We prepared three samples for each HCl concentration and randomly selected five points on each sample for the Raman measurements. Thus, the data points and error bars in Figure 1b were determined with 90 different Raman spectra. As the HCl concentration varied from 0.1 to 10 m, the peak‐shift values increased from 0.14 to 0.93 cm^−1^ for the E_g_‐like peaks and from 0.24 to 1.1 cm^−1^ for the A_g_‐like peaks. To confirm the p‐doping effect on ReSe_2_ once again, we performed KPFM on ReSe_2_ crystal flakes before and after doping with 10 m HCl, as shown in Figure 1c. The KPFM mapping image became darker after doping with the 10 m HCl solution. For quantitative analysis, we extracted the average work‐function values (*Φ*_ReSe2_) from the KPFM mapping images and plotted them, as shown in Figure 1d. *Φ*_ReSe2_ increased by 0.13 eV (from 4.48 to 4.61 eV) after doping with the 10 m HCl solution, and the Fermi‐level shift toward the valence band indicated the p‐type doping effect of HCl on the ReSe_2_. Furthermore, as indicated by the histograms of *Φ*_ReSe2_ shown in Figure 1e, we confirmed that the *Φ*_ReSe2_ values were uniformly distributed near 4.48 and 4.61 eV before and after the 10 m HCl doping, respectively (80% of the pixels were in the range of ±0.3 eV).

**Figure 2:**
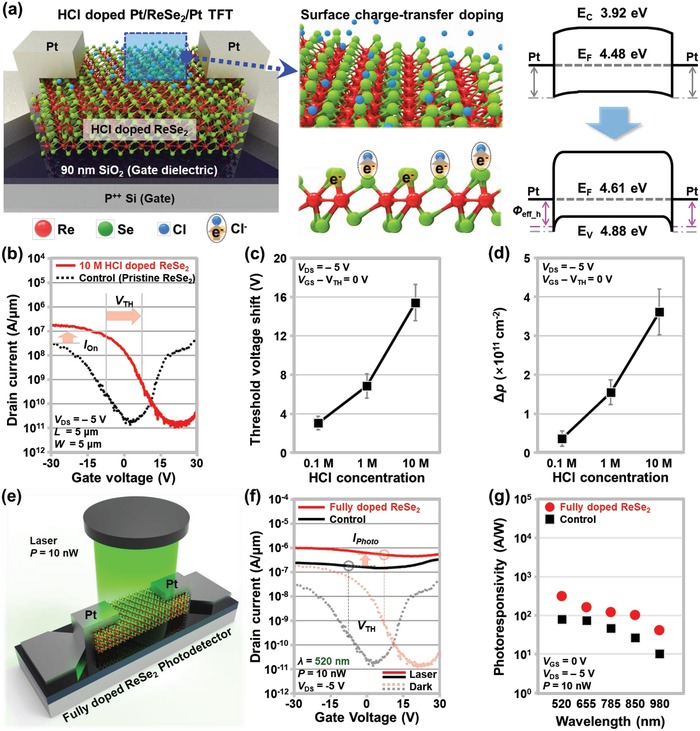
a) Schematics explaining the HCl doping mechanism on the ReSe_2_ surface (left) and energy band diagrams of the Pt/ReSe_2_/Pt junction before and after the HCl p‐doping (right). b) *I*_D_–*V*_G_ characteristic curves of the control and 10 m HCl‐doped ReSe_2_ transistors. c) Extracted threshold‐voltage shift and d) carrier‐concentration increment after the HCl p‐doping with respect to the HCl concentration (at *V*_GS_ = *V*_TH_ and *V*_DS_ = −5 V). e) Schematic of the fully doped ReSe_2_ photodetector under laser illumination. f) *I*_D_–*V*_G_ characteristic curves of the control and fully doped photodetectors in the dark and under laser illumination. g) Photoresponsivity of the control and fully doped photodetectors with respect to the wavelength.

We then fabricated ReSe_2_ transistors and performed electrical measurements on the devices before and after HCl doping to investigate the effects of the doping on the transistor performance. For reliable analysis, we prepared three different ReSe_2_ devices for each HCl doping condition and presented error bars for each data point. **Figure** **2**a shows a schematic of the HCl‐doped ReSe_2_ transistor and the energy band diagrams of the Pt/ReSe_2_/Pt region before and after the HCl doping. The HCl doping is expected to be induced by the surface charge‐transfer phenomenon between the un‐ionized Cl molecules and the ReSe_2_ layer. The un‐ionized Cl molecules have a higher molecular electron affinity than the ReSe_2_ 28 thus, they attract electron carriers from the surface of ReSe_2_. Further experimental proof about the HCl p‐doping based on the Cl molecules is provided in Figure S7 in the Supporting Information. Owing to this electron‐transfer phenomenon, a change in the Fermi level of ReSe_2_ was observed after the HCl doping [4.48 eV → 4.61 eV in the case of 10 m HCl, as shown in Figure 1d]. Consequently, the HCl doping reduced the hole effective barrier height (*Φ*_eff_hole_) at the source Pt/ReSe_2_ junction and increased the hole‐injection probability from the source Pt to the ReSe_2_. This variation in *Φ*_eff_hole_ affected the on‐current (*I*_on_) and threshold voltage (*V*_TH_) of the ReSe_2_ transistor. As shown in Figure 2b, after doping with 10 m HCl, *I*_on_ increased from 27.8 to 97.7 nA µm^−1^ (at *V*_DS_ = −5 V, *V*_G_ = *V*_TH_ − 20 V), and *V*_TH_ was shifted from −8.21 to 7.21 V. The controllability of the HCl doping on the ReSe_2_ transistor was then examined with regard to the threshold‐voltage shift (Δ*V*_TH_ = *V*_TH_HCl_ − *V*_TH_Control_) and the carrier concentration (Δ*p* = Δ*p*_HCl − Δ*p*_Control), as shown in Figure 2c,d, respectively. Here, we applied three HCl concentrations to the devices: 0.1, 1, and 10 m. The Δ*p* was extracted from the *I*_D_–*V*_G_ curves using the following equation: *p* = *I*_D_ *L*/*qWµV*_D_, where *L* and *W* represent the channel length and width, respectively, *q* represents the electron charge, and *µ* represents the mobility of the ReSe_2_ device. As the HCl concentration increased from 0.1 to 10 m, the Δ*V*_TH_ and Δ*p* changed from 3.06 to 15.41 V and from 3.64 × 10^10^ to 3.61 × 10^11^ cm^−2^, respectively. These p‐doping concentrations were comparable to the reported values for p‐doping techniques: from 2.1 × 10^11^ to 5.2 × 10^11^ cm^−2^ (OTS on WSe_2_),^25^ ≈2.2 × 10^12^ cm^−2^ (FOTS doping on MoS_2_),^26^ ≈10^10^ cm^−2^ (M‐DNA on MoS_2_ and WSe_2_),^29^ 5.25 × 10^12^ cm^−2^ (O_2_ plasma treatment on ReS_2_),^30^ ≈2.2 × 10^12^ cm^−2^ (NO_2_ doping on WSe_2_),^31^ and 1.67 × 10^11^ to 1.32 × 10^12^ cm^−2^ (HCl doping on WSe_2_).^28^ The ratio values of *I*_ON_ and *µ*_FE_ after/before the p‐doping increased from 2.05 to 3.52 and from 2.55 to 4.37, respectively (Figure S1, Supporting Information). Electrical measurements were performed again in the dark and under laser illumination. As shown in Figure 2e, a 520 nm laser beam with 10 nW power was applied to the ReSe_2_ channel region. The fully doped ReSe_2_ device exhibited a higher photocurrent than the control ReSe_2_ device in the entire gate‐voltage region. Here, “fully doped ReSe_2_” indicates that the whole opened area of ReSe_2_ was doped (Figure 2f). The photocurrent of the 10 m HCl‐doped ReSe_2_ device was 5.25 × 10^−7^ A µm^−1^ at *V*_GS_ = *V*_TH_, and that of the control device was 1.86 × 10^−7^ A µm^−1^. This enhancement can be explained by the reduction in the recombination rate due to the Cl passivation of defect sites in ReSe_2_.^32^ The photocurrent of the fully doped device was also higher than that of the control device over the entire wavelength region of 520–980 nm, as shown in Figure 2g. As a result, the photoresponsivity of the fully doped and control ReSe_2_ devices was distributed between 314.4 and 41.96 A W^−1^ and between 79.99 and 10.29 A W^−1^, respectively.

**Figure 3:**
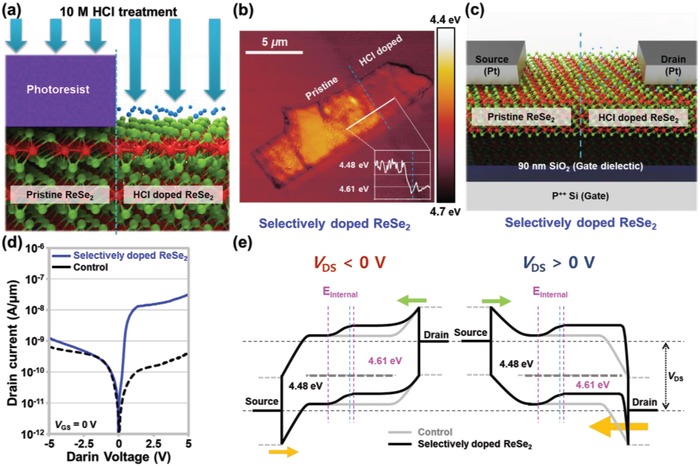
a) Schematic showing the selective HCl doping process applied to the ReSe_2_ channel. b) KPFM mapping image and work‐function profile (inset) obtained on the surface of selectively HCl‐doped ReS_2_. c) Schematic of the selectively doped ReSe_2_ transistor. d) *I*_D_–*V*_D_ characteristic curves of the control and selectively doped ReSe_2_ devices. e) Energy band diagrams of the control and selectively doped Pt/ReSe_2_/Pt junctions under negative and positive *V*_DS_ conditions.

To improve the photoresponsivity further, we then formed an internal electric field in the middle of the ReSe_2_ channel by using selective HCl doping instead of full HCl doping. **Figure** **3**a illustrates the selective HCl doping process, where part of the channel was covered with photoresist and the remaining part was exposed to the 10 m HCl. The ReSe_2_ homojunction formed via selective HCl doping was examined through KPFM mapping, as shown in Figure 3b. The right side of the blue dotted line was exposed to the 10 m HCl solution, and we found that the work function in this region increased from 4.48 to 4.61 eV. The work function changed gradually and linearly, with a transition width of ≈0.65 µm (12 out of 87 points: 58.6 nm per interval) at the boundary of the pristine and HCl‐doped regions. This indicates that the internal electric field was formed in the boundary region of 0.65 µm owing to the difference in the carrier concentration. A schematic of the selectively doped Pt/ReSe_2_/Pt device structure with a back‐gate electrode is presented in Figure 3c. To clarify the sign of the applied bias and the direction of the current, we defined the electrode on pristine ReSe_2_ as the source and the electrode on HCl‐doped ReSe_2_ as the drain. Figure 3d shows the *I*_D_–*V*_D_ characteristics of the control and selectively doped ReSe_2_ devices, where *V*_GS_ is 0 V. In the control device, similar current levels were observed in the negative and positive drain voltage regions. In the selectively doped ReSe_2_ device, a higher current was observed in the positive‐voltage region (32.7 nA µm^−1^ at *V*_DS_ = 5 V and 1.26 nA µm^−1^ at *V*_DS_ = −5 V). This is because the carrier injection at the ReSe_2_/Pt drain junction was enhanced by the selective doping. Figure 3e shows the energy band structures of the Pt/ReSe_2_/Pt junctions formed on the selectively doped ReSe_2_, which were drawn under different drain bias conditions (left: *V*_DS_ < 0 V; right: *V*_DS_ > 0 V). In the case of a negative drain voltage, because high barriers were initially formed at the both Pt/ReSe_2_ (source side) and ReSe_2_/Pt (drain side) junctions, no reduction in the drain current was observed, even though an increase in the effective hole barrier height was predicted due to the selective doping. In contrast, under the positive drain bias condition, the hole‐carrier injection at the Pt/WSe_2_ drain junction was expected to increase significantly owing to the selective doping of ReSe_2_. This manifested as an increase in the drain current by more than two orders of magnitude. Additionally, we provide the energy band diagrams of the control, fully doped, and selectively doped Pt/ReSe_2_/Pt junctions under different drain bias conditions in Figure S2 in the Supporting Information.

**Figure 4:**
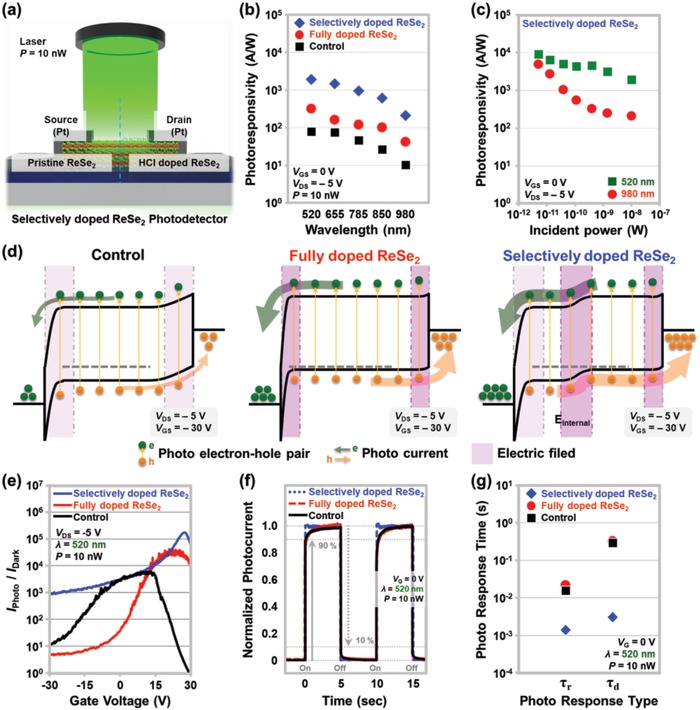
a) Schematic of the selectively doped ReSe_2_ phototransistor under laser illumination. Photoresponsivity with respect to b) the wavelength and c) the incident laser power. d) Energy band diagrams of the control, fully doped, and selectively doped ReSe_2_ photodetectors under *V*_GS_ = −30 V and *V*_DS_ = −5 V. e) *I*_Photo_/*I*_Dark_ ratios of the control, fully doped, and selectively doped ReSe_2_ photodetectors. f) Comparison of the normalized temporal photoresponse curves and g) extracted rise and decay times for the control, fully doped, and selectively doped ReSe_2_ photodetectors.

Finally, the performance of control, fully doped, and selectively doped ReSe_2_ photodetectors was evaluated, as shown in **Figure** **4**. Figure 4a presents a schematic of the photodetector fabricated on the selectively doped ReSe_2_. We applied lasers with power of 10 nW and wavelengths between 520 and 980 nm to the three types of devices and measured the photocurrents. The selectively doped ReSe_2_ device exhibited a higher photocurrent than the control ReSe_2_ device by at least one order of magnitude, and the photocurrent was higher than that of the fully doped ReSe_2_ device (Figure S3, Supporting Information). Under laser illumination of λ = 520 nm, the photocurrent values (*V*_GS_ = 0 V) for the control, fully doped, and selectively doped ReSe_2_ devices were 1.61 × 10^−7^, 6.32 × 10^−7^, and 3.95 × 10^−6^ A µm^−1^, respectively. We then obtained the photoresponsivity values at the bias point of *V*_GS_ = 0 V and *V*_DS_ = −5 V in the *I*_Photo_ versus *V*_GS_ characteristic curves, which are plotted with respect to the wavelength in Figure 4b. In the case of the selectively doped ReSe_2_ device, relatively high photoresponsivity values appeared between 1.93 × 10^3^ (λ = 520 nm) and 208.8 (λ = 980 nm) A W^−1^. The photoresponsivity values of the control and fully doped devices were distributed between 79.99 and 10.29 and between 314.4 and 41.96, respectively, in the same wavelength range. Figure 4c shows the photoresponsivity of the selectively doped ReSe_2_ device under two wavelength conditions, which was plotted with respect to the incident laser power. The highest photoresponsivity values under each wavelength condition were obtained at the lowest power of 5.02 pW: 9.1 × 10^3^ and 4.96 × 10^3^ A W^−1^ at λ = 520 and 980 nm, respectively. Overall, a higher photoresponsivity was achieved at a lower laser power. This inverse relationship between the photoresponsivity and the incident laser power was due to the suppression of the scattering of photocarriers under the low‐power condition.^33^, ^34^, ^35^ Figure 4d shows the energy band structures of control, fully doped, and selectively doped ReSe_2_ devices at *V*_DS_ = −5 V and *V*_GS_ = −30 V. As previously mentioned, by applying the HCl doping to the entire ReSe_2_ region, a higher photocurrent was obtained, but a higher dark current was observed owing to the increased hole‐injection probability. As shown in the rightmost diagram, the selective HCl doping applied to the right‐half region of ReSe_2_ improved the photocarrier collection by forming an internal electric field at the p^+^/p junction boundary and also maintained the hole‐carrier injection from the source. In addition, the defects on the ReSe_2_ surface were expected to be passivated by the HCl treatment. This appeared to reduce the recombination rate in ReSe_2_ and to increase photocarrier lifetime and photoresponsivity.

In addition to the photoresponsivity, the dark‐current level is important for evaluating the performance of photodetectors. Thus, we investigated the *I*_Photo_/*I*_Dark_ ratios of the fully and selectively doped ReSe_2_ devices (Figure 4e). As shown in Figure S3 in the Supporting Information, the dark current was significantly reduced and a high photocurrent remained when a negative drain voltage was applied to the selectively doped device, compared with the case of a positive drain voltage. Therefore, we obtained the *I*_Photo_/*I*_Dark_ values from the characteristic curves under *V*_DS_ = −5 V for the control, fully doped, and selectively doped devices, which were then plotted with respect to the gate voltage. As the gate voltage changed from −30 to 30 V, the *I*_Photo_/*I*_Dark_ initially increased and then decreased for all types of the devices. This is because the effective hole barrier height at the source‐side Pt/ReSe_2_ junction first increased (a dark current decreased), and then the effective electron barrier height at the drain‐side ReSe_2_/Pt junction decreased (a dark current increased). The control, fully doped, and selectively doped ReSe_2_ devices exhibited maximum *I*_Photo_/*I*_Dark_ values of 6.24 × 10^3^, 4.31 × 10^4^, and 1.75 × 10^5^ at the gate voltages of 12.7, 20.8, and 28.1 V, respectively, where the minimum dark currents appeared. The *I*_Photo_/*I*_Dark_ of the selectively doped device was superior to those of the other devices over the entire gate‐voltage region owing to the lower dark current and higher photocurrent of this device. Finally, the time‐dependent photoresponse characteristic was investigated for the three types of ReSe_2_ photodetectors. Figure 4f shows the temporal photoresponse curves obtained under irradiation with laser pulses having a wavelength of 520 nm and power of 10 nW, which were normalized by the maximum photocurrent values. The photocurrent of the selectively doped device reached its maximum value (on‐state) and returned to its initial dark‐current level (off‐state) more rapidly than the control and fully doped devices. This faster photoresponse was observed in every laser on/off cycle. For more accurate comparison, we extracted the rise (τ_r_) and decay (τ_d_) times at 20 different rising and decaying edges, which were plotted as shown in Figure 4g. The standard errors for the rise and decay times were within 5%. The time required to increase from the dark‐current level to 90% of the maximum photocurrent was defined as the rise time, and the time required to decrease from the maximum photocurrent to 10% of the maximum value was considered as the decay time. The extracted rise and decay times (τ_r_ and τ_d_) were 10.5 and 291 ms, respectively, for the control ReSe_2_ device; 17 and 323 ms, respectively, for the fully doped ReSe_2_ device; and 1.4 and 3.1 ms, respectively, for the selectively doped ReSe_2_ device. As previously mentioned, the selectively doped ReSe_2_ photodetector exhibited the fastest response to the laser pulses, and the response of the control ReSe_2_ device was slightly faster than that of the fully doped device. The selective doping formed an electric field at the interface between the doped and undoped ReSe_2_ regions, enhancing the splitting of the photogenerated electron–hole pairs. However, HCl doping of the entire ReSe_2_ region narrowed the depletion width at the Pt/ReSe_2_ junctions compared with the control device, hindering the photocarrier splitting and thereby increasing the photoresponse time.

## 3 Conclusion

We simultaneously enhanced the photo and temporal responses of the ReSe_2_ photodetector for detecting 520−980 nm wavelength light by using an HCl‐based selective p‐doping technique (refer to Table S1, Supporting Information). The effect of p‐type doping on the ReSe_2_ material was experimentally investigated via Raman spectroscopy and KPFM. Because the p‐doping of ReSe_2_ was based on the charge transfer phenomenon from the un‐ionized Cl molecules to the ReSe_2_ surface, by adjusting the concentration of the HCl solution (0.1, 1, and 10 m), we controlled the doping concentration (Δ*p*) of the ReSe_2_ between 3.64 × 10^10^ and 3.61 × 10^11^ cm^−2^. We subsequently confirmed that the *V*_TH_, *µ*_FE_, *I*_ON_, and photoresponsivity of the ReSe_2_ devices changed as the doping concentration increased in the Δ*p* range: the Δ*V*_TH_ increased from 3.06 to 15.41 V, the ratio of *I*_ON_ after/before the doping increased from 2.05 to 3.52, the ratio of *µ*_FE_ after/before the doping increased from 2.55 to 4.37, and the photoresponsivity at 520 nm increased from 79.99 to 314.4 A W^−1^. Using the selective HCl p‐doping process, the key performance indices of the ReSe_2_ photodetector were improved simultaneously, compared with the control ReSe_2_ photodetector. The photoresponsivity increased from 79.99 to 1.93 × 10^3^ A W^−1^, and the rise and decay times were enhanced from 10.5 to 1.4 ms and from 291 to 3.1 ms, respectively, where *V*_GS_ was 0 V and *V*_DS_ was −5 V. This is because the p/p^+^ junction formed via selective HCl p‐doping of the ReSe_2_ channel region significantly affected the splitting of photocarriers and the injection of carriers from Pt to ReSe_2_. The proposed p‐doping technique and its fundamental analysis provide a scientific foundation for implementing high‐performance TMD‐based electronic and optoelectronic devices.

## 4 Experimental Section

*HCl p‐Doping of ReSe_2_*: 0.1, 1, and 10 m HCl aqueous solutions were prepared by mixing saturated HCl aqueous solution (37% or 12 m) and deionized water. ReSe_2_ samples were then dipped into the prepared HCl solutions to dope ReSe_2_. After 5 min, the ReSe_2_ samples were rinsed with deionized water to remove residual of the HCl solution on the surface of ReSe_2_.

*Raman and KPFM Analyses of Fully and Selectively HCl‐Doped ReSe_2_*: The HCl‐doped ReSe_2_ samples were analyzed through Raman spectroscopy, (Alpha300 M+, WITec), and KPFM measurements. Raman spectroscopy with an excitation wavelength of 532 nm was used, where the laser beam diameter was ≈0.7–0.9 µm, the instrumental spectral resolution was less than 0.9 cm^−1^, and the integration time was 5 s. For the KPFM measurement, a platinum/iridium (Pt/Ir)‐coated Si tip was used and the tip was calibrated on a highly oriented pyrolytic graphite (HOPG) surface. The surface work function of the samples was obtained from the contact potential difference (CPD) between the tip and the HOPG work function (*Φ*_tip_ – *Φ*_HOPG_ = *V*_CPD_), where the standard work function value of HOPG was used (4.6 eV). Further details on the KPFM analysis are provided in Figure S8 in the Supporting Information.

*Fabrication of the Control, Fully HCl‐Doped, and Selectively HCl‐Doped Devices*: The ReSe_2_ crystals were commercially purchased and 35 nm thick ReSe_2_ layer was mechanically exfoliated to the 90 nm thick SiO_2_ on a heavily boron‐doped Si substrate by using adhesive tape (224SPV, Nitto). The remaining tape residue was then removed with acetone. For the control device, source/drain electrode regions were patterned by optical lithography (channel length and width: both 5 µm). Then, 10 nm platinum (contact metal) and 40 nm palladium (pad metal) layers were deposited in an e‐beam evaporation system. In case of selectively HCl‐doped ReSe_2_ devices, additional optical lithography process was conducted to block half of the ReSe_2_ region from HCl doping. After the selective HCl doping, the photoresist pattern was removed with acetone.

*Characterization of the ReSe_2_ Transistor Devices*: The control and fully doped ReSe_2_ photodetector devices were electrically investigated through current–voltage measurements (*I*_D_–*V*_G_ and *I*_D_–*V*_D_) by a Keysight B2912A precision source/measure unit. The threshold voltage (*V*_TH_), carrier concentration (Δ*p*), and field‐effect mobility (*µ*_FE_) were calculated from the measured data, where all drain currents (*I*_D_) were normalized by the channel width (5 µm). The carrier concentration and field‐effect mobility were respectively extracted using *p* = *I*_D_ *L*/*qWµV*_D_ and *µ*_FE_ = *L*/(*WV*_D_ *C*_OX_) × (∂*I*_D_/∂*V*_G_), where *L* and *W* are the length and width of the channel, *q* is the electron charge, and *C*_OX_ is the capacitance of the gate oxide, respectively.

*Characterization of the ReSe_2_ Photodetector Devices*: The control, fully doped, and selectively doped ReSe_2_ photodetector devices were investigated through the electrical measurement (*I*_D_–*V*_G_) under dark and illuminated conditions. The light sources were dot lasers with wavelengths of 520, 655, 785, 850, and 980 nm. The photoresponsivity (*R*) was calculated by the relationship, *R* = *I*_Photo_/*P*_Light_, where *I*_Photo_ is the generated photocurrent and *P*_Light_ is the total incident optical power. The photoresponse speed was analyzed under 520 nm laser illumination, which was turned on for 5 s and then off for another 5 s (1 cycle was 10 s).

## Conflict of Interest

The authors declare no conflict of interest.

## Supporting information

Supplementary
