# Supplementary material for: Rhenium Diselenide (ReSe2) Near‐Infrared Photodetector: Performance Enhancement by Selective p‐Doping Technique
Source: Adv Sci (Weinh). 2019 Aug 27;6(21):1901255. doi: 10.1002/advs.201901255 (PMC6839648; doi:10.1002/advs.201901255)
Supplement: Supplementary file 1 — Supplementary [file ADVS-6-1901255-s001.pdf]

Copyright WILEY-VCH Verlag GmbH & Co. KGaA, 69469 Weinheim, Germany, 2019.

ADVANCED SCIENCE Open Access

Supporting Information

for *Adv. Sci.,* DOI: 10.1002/advs.201901255

Rhenium Diselenide ($ReSe_2$) Near-Infrared Photodetector: Performance Enhancement by Selective p-Doping Technique

*Jinok Kim, Keun Heo, Dong-Ho Kang, Changhwan Shin, Sungjoo Lee, Hyun-Yong Yu, and Jin-Hong Park**

Copyright WILEY-VCH Verlag GmbH & Co. KGaA, 69469 Weinheim, Germany, 2019.

Supporting Information

Rhenium Di-Selenide ($ReSe_2$) Near-IR Photodetector: Performance Enhancement by Selective p-Doping Technique

*Jinok Kim, Keun Heo, Dong-Ho Kang, Changhwan Shin, Sungjoo Lee, Hyun-Yong Yu, and Jin-Hong Park**

J. Kim, Dr. K. Heo, Dr. D.-H. Kang, Prof. C. Shin, Prof. J.-H. Park
Department of Electrical and Computer Engineering, Sungkyunkwan University, Suwon 16419, Korea
E-mail: jhpark9@skku.edu
Dr. D.-H. Kang
School of Electrical and Electronic Engineering, Nanyang Technological University, 50 Nanyang Avenue, 639798 Singapore, Singapore
Prof. S. Lee
SKKU Advanced Institute of Nano Technology (SAINT), Sungkyunkwan University, Suwon 16419, Korea
Prof. H.-Y. Yu
School of Electrical Engineering, Korea University, Seoul 02841, Korea

Thickness of the used $ReSe_2$ flake. I_{ON} and μ_{FE} ratio between control and HCl-doped $ReSe_2$ devices with respect to different HCl concentrations

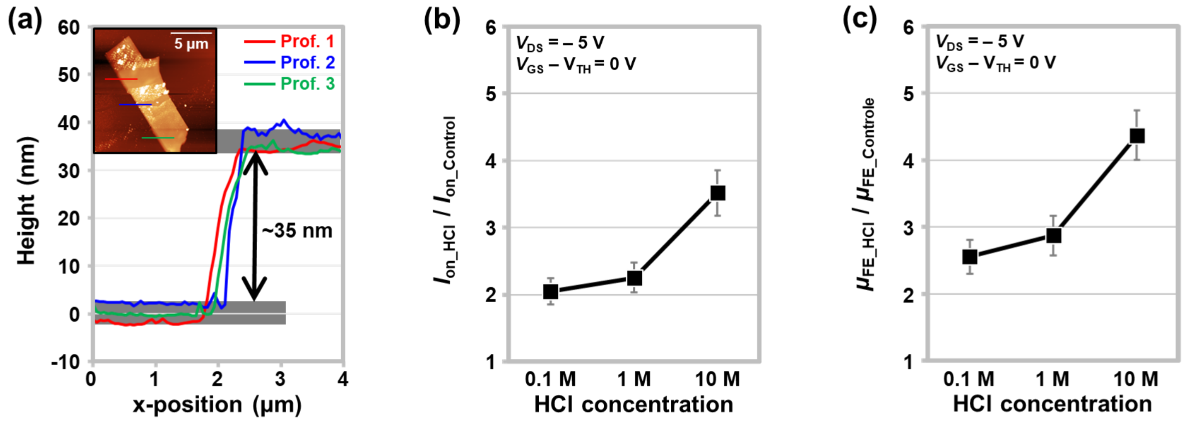

Figure S1. (a) The AFM image and depth profiles of HCl-doped $ReSe_2$ on SiO_2. The ratio of (b) I_{ON} and (c) field effect mobility between control and fully doped $ReSe_2$ devices under V_{DS} = –5 V & V_{GS} = V_{TH} with respect to HCl concentration.

$ReSe_2$ flakes with thickness of approximately 35 nm were used for all experiments. The thickness was confirmed by AFM measurement. The on-current and μ_{FE} ratio under V_{DS} = –5 V and V_{GS} = V_{TH} were improved from 2.05 to 3.52 and 2.55 to 4.37, respectively, by increasing the HCl concentration from 0.1 to 10 M.

Energy band diagrams of the control, fully doped, and selectively doped Pt/$ReSe_2$/Pt junctions under different drain bias condition. (V_{DS} = 0, 5, -5 V and V_G = 0 V)

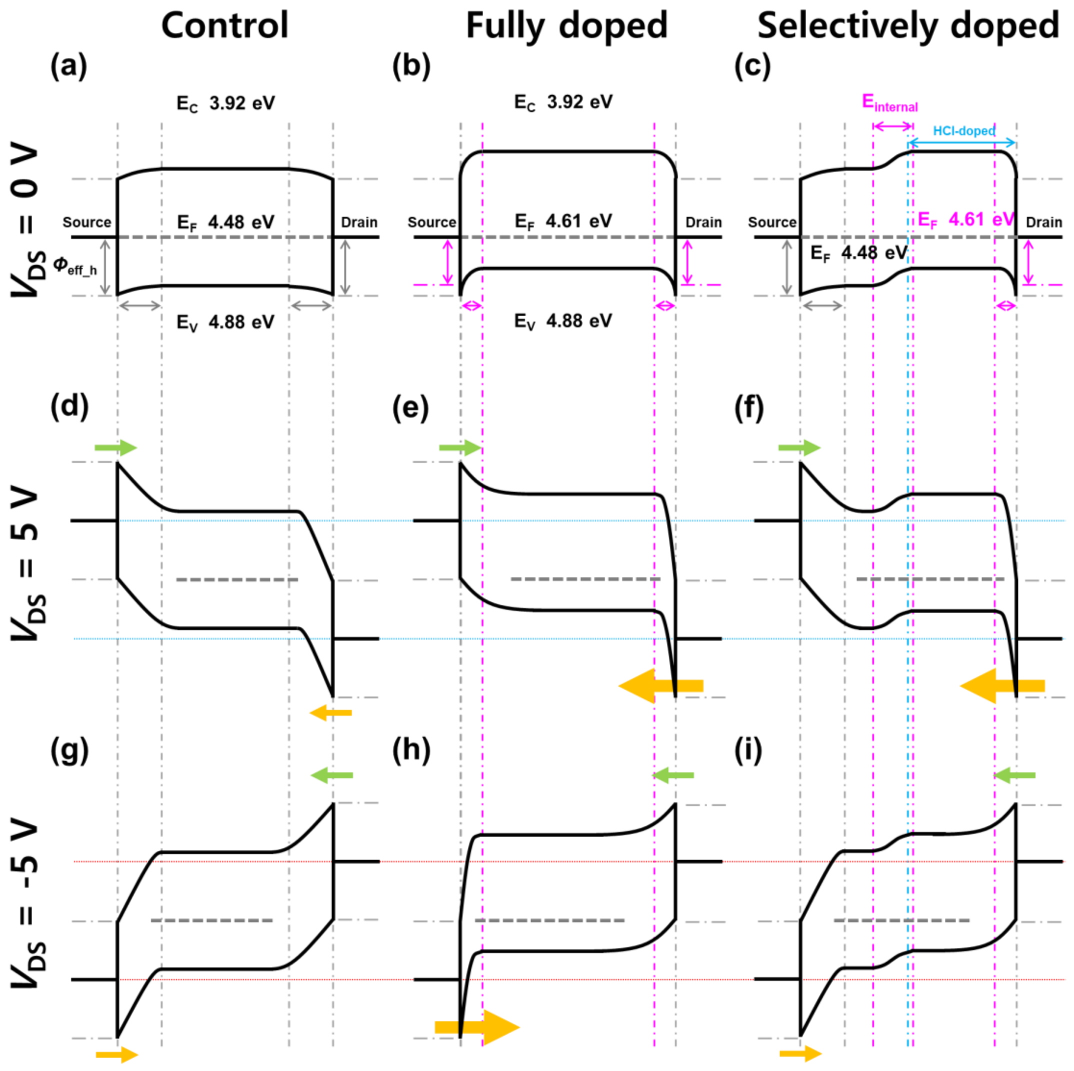

Figure S2. Energy band diagrams of the control, fully doped, and selectively doped Pt/$ReSe_2$/Pt junctions under V_G = 0 V: (a) control (undoped), (b) fully doped, and (c) selectively doped junctions under V_{DS} = 0 V (equilibrium). (d) control, (e) fully doped and (f) selectively doped $ReSe_2$ junctions under V_{DS} = 5 V. (g) control, (h) fully doped and (i) selectively doped $ReSe_2$ junctions under V_{DS} = –5 V.

In the fully doped $ReSe_2$ device, the drain current (= dark current) increases, compare to the control device under both 5 V and –5 V of V_{DS}, because the HCl doping applied on the entire $ReSe_2$ region decreases the effective hole barrier height at both source and drain Pt-$ReSe_2$ junctions. However, in the selectively doped $ReSe_2$ device, the effective hole barrier height at the source Pt-$ReSe_2$ junction is not changed even after the HCl doping, consequently maintaining a drain current (= dark current) as low as that of the control device under V_{DS} = –5 V.

Operation of the selectively doped $ReSe_2$ photodetector with respect to negative and positive drain voltages.

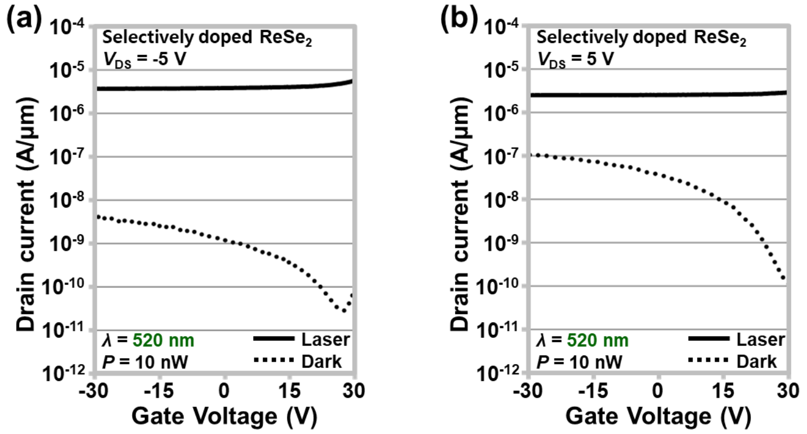

Figure S3. I_D–V_G characteristic curves of the selectively doped $ReSe_2$ devices measured under dark and laser illuminated conditions under the drain biases of (a) –5 V and (b) 5 V (Laser irradiation power: 10 nW, wavelength: 520 nm).

A dark current was considerably reduced and a high photocurrent was remained when a negative drain voltage was applied to the selectively doped $ReSe_2$ device, compared to the case under a positive drain voltage.

Energy band diagrams of the control, fully doped, and selectively doped $Pt/ReSe_2/Pt$ junctions under different gate bias condition. (V_G= 30, 0, -30 V and V_{DS} = -5 V)

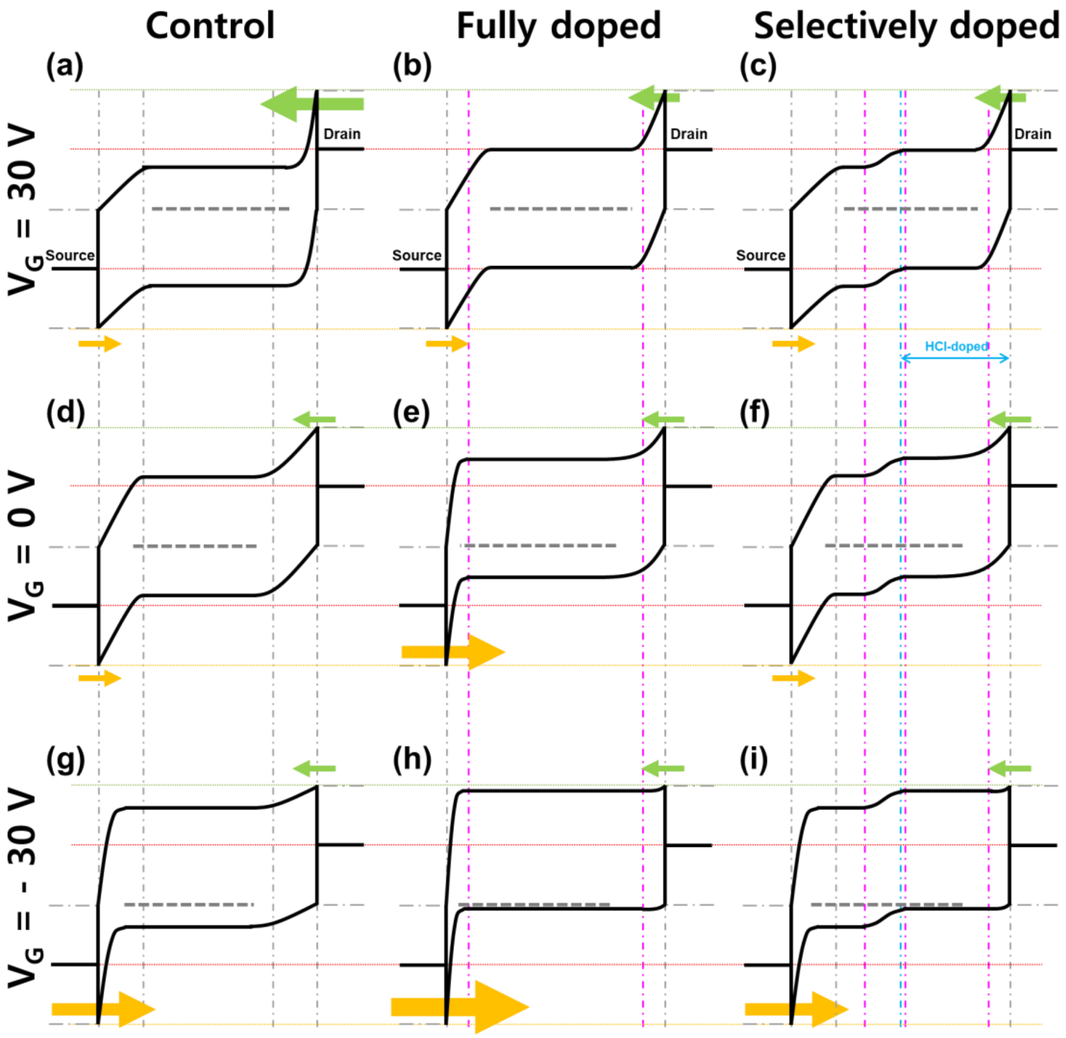

Figure S4. Energy band diagrams of the control, fully doped, and selectively doped $Pt/ReSe_2/Pt$ junctions under V_{DS} = –5 V: (a) control (undoped), (b) fully doped, and (c) selectively doped junctions under V_G = 30 V. (d) control, (e) fully doped and (f) selectively doped $ReSe_2$ junctions under V_G = 0 V. (g) control, (h) fully doped and (i) selectively doped $ReSe_2$ junctions under V_G = – 30 V.

For all types of $ReSe_2$ devices under a negative drain bias of –5 V, the effective hole barrier heights at the source-$ReSe_2$ and $ReSe_2$-drain junctions decrease at –30 V and at 30 V of V_G, respectively. Thus, the dark current changes greatly depending on the V_G. However, in the case of selectively doped device (Figure S4c, f, i), the effect of V_G on internal electric field is negligible. Thereby, the photocurrent of selectively doped $ReSe_2$ device, which is predicted to be mainly determined by the internal electric field, is consistent regardless of V_G (refer to the Figure S3a).

Performance Comparison of various 2D-material-based photodetectors.

Table S1.
In this study, by applying a selective HCl p-doping technique, we achieved a $ReSe_2$ photodetector having long-wavelength detection, a short response time, and a high photoresponsivity, simultaneously

Doping Method	Material	Wavelength [nm]	Incident-power [W]	Responsivity [A/W] Before	Responsivity [A/W] After	Temporal response Rise time / Decay time [s] Before	Temporal response Rise time / Decay time [s] After	Ref.
HCl doping	$ReSe_2$	520	10 nW	7.99×10^{1}	3.14×10^{2} ↑	1.05×10^{-2} / 2.91×10^{-1}	1.7×10^{-2} / 3.23×10^{-1} ↑	***This work***
**Selective HCl doping**					**1.93×10^{3}** ↑		**2.7×10^{-3} / 3.1×10^{-3}** ↓	
PPh_3 doping	MoS_2	520	1 nW	1.81×10^{3}	6.46×10^{4} ↑	6.97 / 12.2	8.31 / 14.1 ↑	Adv. Mater. **2016**, 28, 6711
PPh_3-APTES doping					5.63×10^{4} ↑		9.11 / 15.3 ↑	
PPh_3 doping	$ReSe_2$			1.68×10^{2}	6.54×10^{2} ↑	1.0×10^{-2} / 4.1×10^{-2}	3.0×10^{-2} / 6.4×10^{-2} ↑	
PPh_3-APTES doping					1.73×10^{4} ↑		5.8×10^{-2} / 2.63×10^{-1} ↑	
PPh_3 doping	WSe_2	520	1 nW	1.86×10^{2}	8.63×10^{3} ↑	3.82×10^{-2} / 1.91×10^{-1}	9.7×10^{-2} / 8.27×10^{-1} ↑	Adv. Mater. **2016**, 28, 4824
O_2 plasma doping and thinning	ReS_2	405	5 pW	2.5×10^{7}	2.5×10^{7} –	16.7 / 25.2	6.7×10^{-1} / 5.6 ↓	Adv. Mater. **2016**, 28, 6985
APTMS doping	$ReSe_2$	785	40 nW	1.4×10^{1}	7.17×10^{1} ↑	1.3×10^{-3} / 2.7×10^{-3}	1.9×10^{-3} / 4.5×10^{-3} ↑	Org. Electron. **2018**, 53, 14

Responsivity: ↑ Improve, ↓ Degrade, Temporal response: ↓ Improve, ↑ Degrade,

Temporal photoresponse curves under a 520-nm laser for 20 cycles.

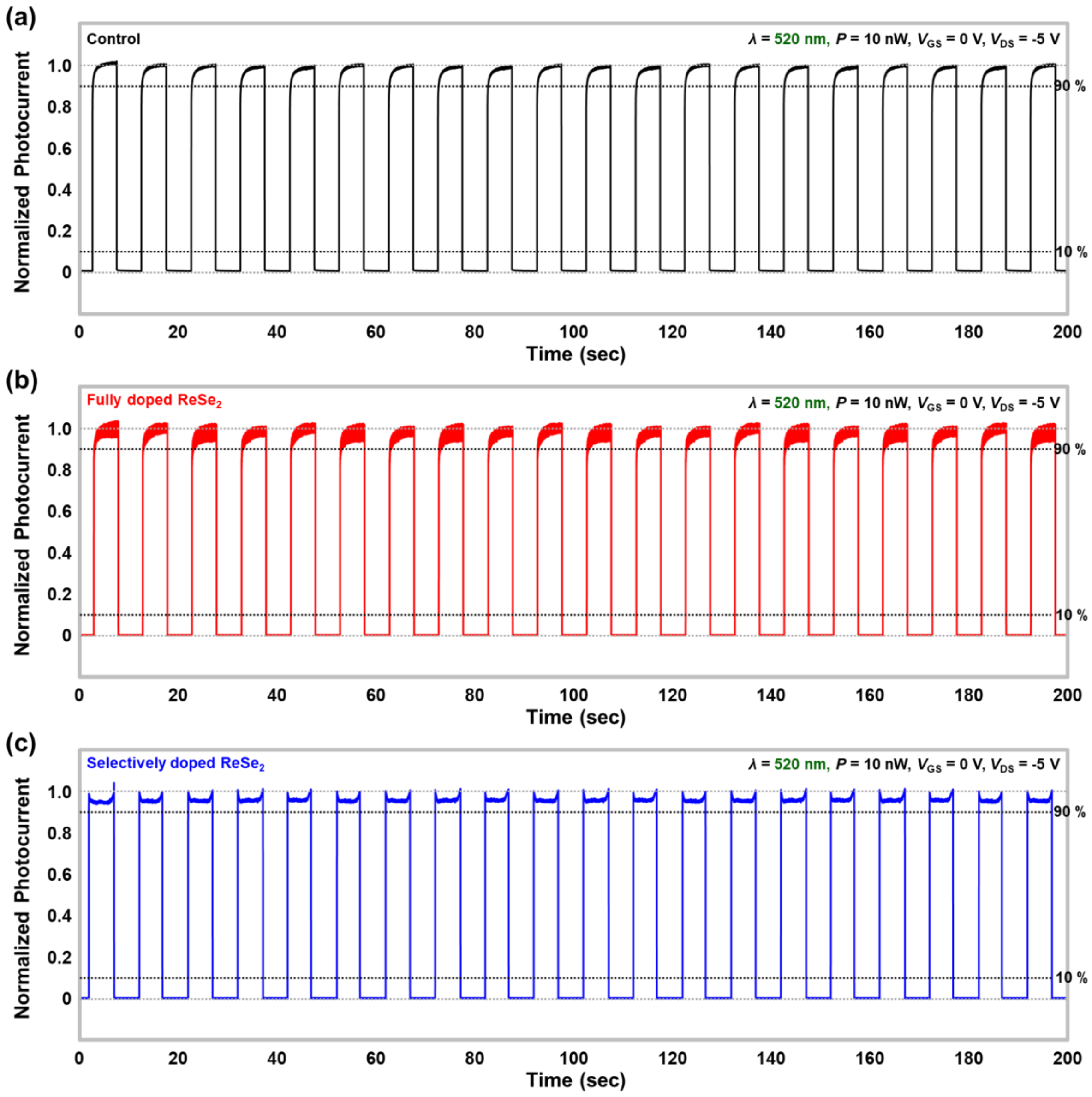

Figure S5. Normalized temporal photoresponse curves of (a) control, (b) fully doped, and (c) selectively doped $ReSe_2$ devices under the 520-nm laser for 20 cycles.

Temporal photoresponse curves under a 980-nm laser for 20 cycles.

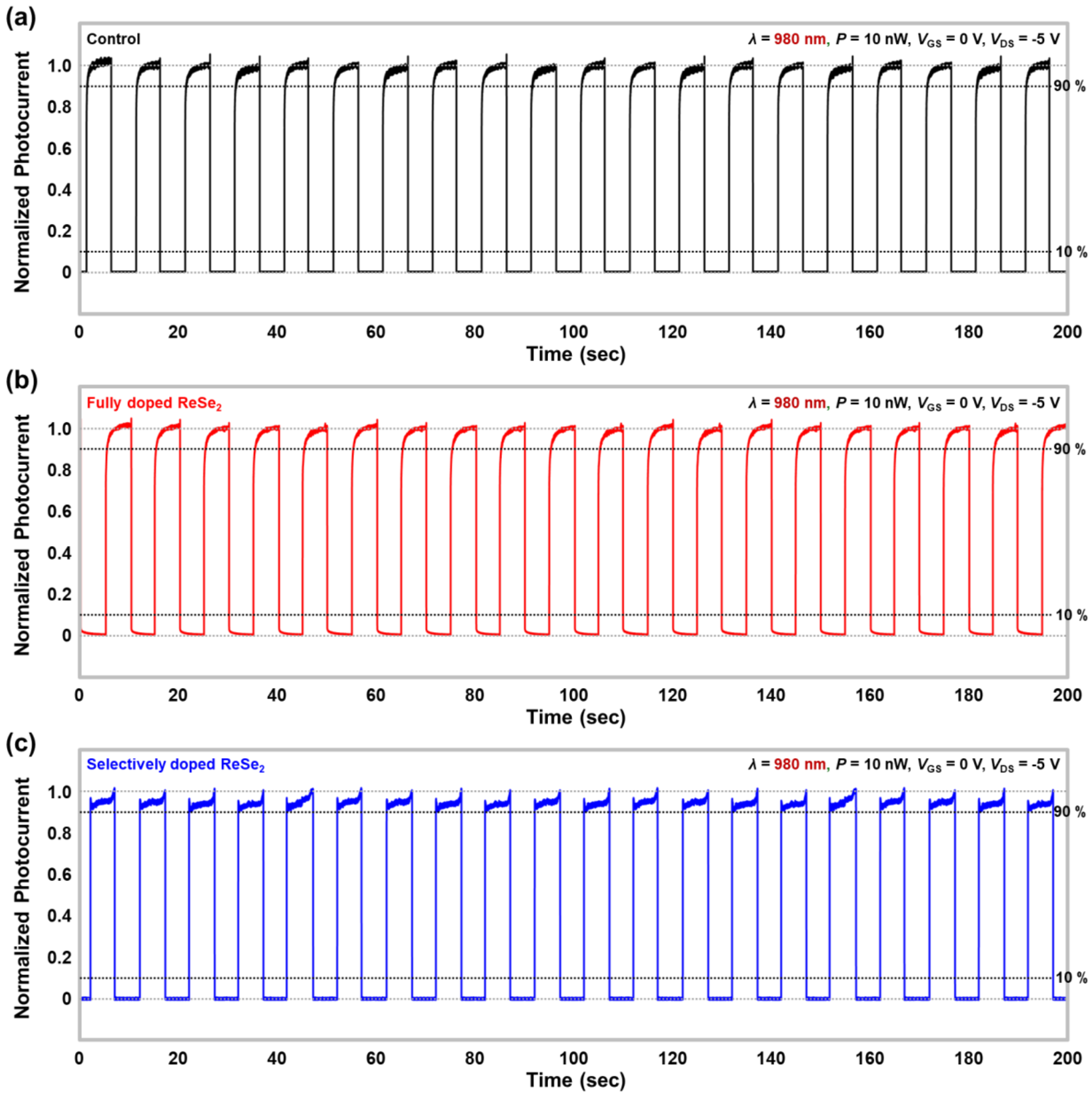

Figure S6. Normalized temporal photoresponse curves of (a) control, (b) fully doped, and (c) selectively doped $ReSe_2$ devices under 980-nm laser for 20 cycles.

Micro-XPS and electrical measurements in HCl and NaCl treated $ReSe_2$ device samples.

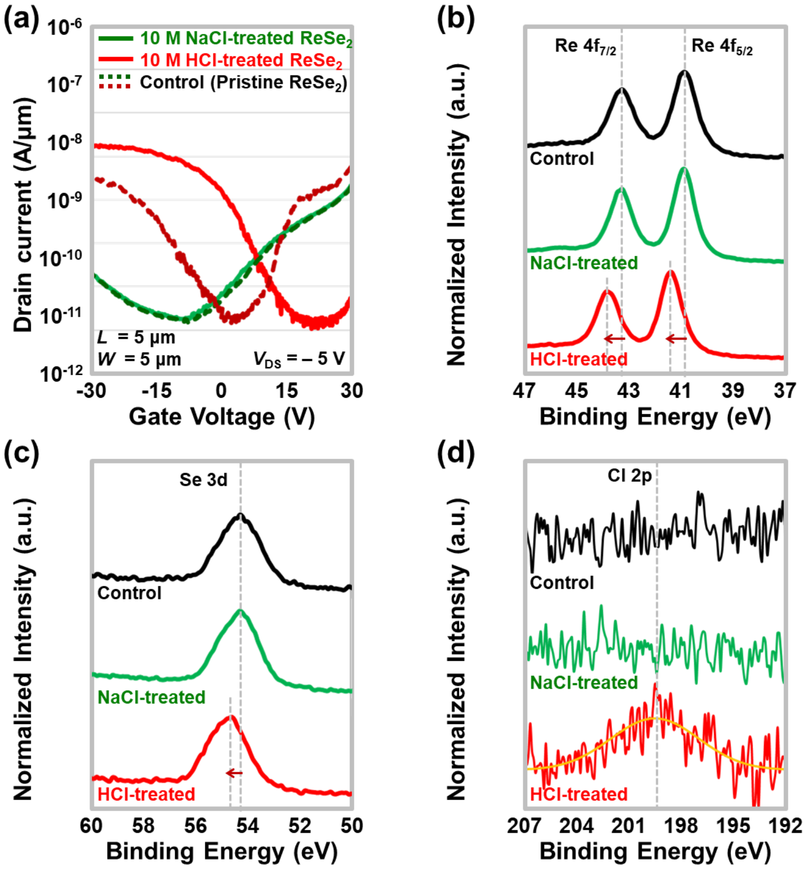

Figure S7. (a) I_D-V_G characteristic curves of control, 10M NaCl-treated, and 10M HCl-treated $ReSe_2$ devices. XPS analysis of control, 10 M NaCl-treated, and 10M HCl-treated $ReSe_2$ device samples: binding energy peaks of (b) Re $4f_{7/2}$ and $4f_{5/2}$, (c) Se 3d, and (d) Cl 2p.

In contrast to the HCl-treated $ReSe_2$ device, the NaCl-treated $ReSe_2$ device showed a similar I_D-V_G characteristic curve to that of the control device (Figure S7a). As shown in Figures S7b and S7c, the positions of Re $4f_{7/2}$, Re $4f_{5/2}$, and Se 3d peaks were not changed after the NaCl treatment, compared to the values of the control sample. Whereas, the peaks were positively shifted by 0.5, 0.5, and 0.3 eV after the HCl treatment, respectively. Note that all XPS peaks were normalized by C1s peak (284.4 eV).

We subsequently investigated the Cl 2p peak for the three kinds of $ReSe_2$ samples, which was predicted to be the cause of the p-doping. As shown in Figure S7d, the Cl 2p peak (at 199.5 eV) was confirmed only on the HCl-treated $ReSe_2$ sample, not on the NaCl-treated $ReSe_2$. This indicates that the Cl molecules attached on $ReSe_2$ surface were responsible for the p-doping phenomenon.

KPFM analysis of pristine and 10 M HCl-doped $ReSe_2$

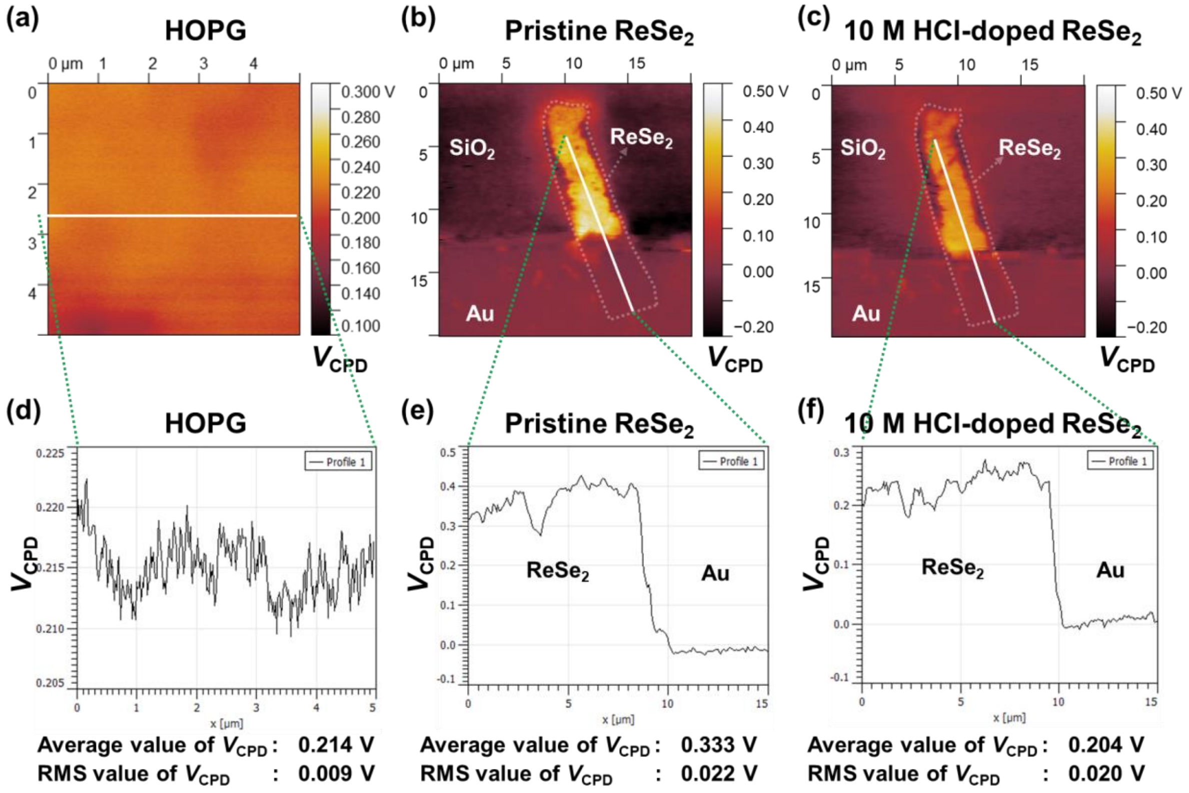

Figure S8. KPFM mapping images taken on the surfaces of (a) HOPG and (b-c) $ReSe_2$ flakes before and after the HCl treatment. (d–f) The corresponding contact potential difference (V_{CPD}) values.

We extracted the work function of the KPFM probe tip using freshly cleaved, highly oriented pyrolytic graphite (HOPG, work function: 4.6 eV) and then estimated the $ReSe_2$ work function. As seen in Figures S8a and S8d, the contact potential difference (V_{CPD}) between the tip and the HOPG was approximately 0.21 V. Consequently, the work function of the tip was calculated to be 4.81 eV.

$$V_{CPD} = \Phi_{tip} - \Phi_{sample}$$

$$\Phi_{tip} = \Phi_{HOPG} + 0.21\ \mathrm{V} = 4.81\ \mathrm{eV}$$

To reconfirm this tip work function value, we extracted the V_{CPD} value of an Au electrode, which was confirmed as 0 V (Figure S8e and f).

$$0\ \mathrm{V} = \Phi_{tip} - \Phi_{Au}$$

The V_{CPD} values of pristine and HCl-doped $ReSe_2$ extracted from Figures S8e and S8f were 0.33 and 0.2 V, respectively. With the V_{CPD} values, the work function of $ReSe_2$ was quantitatively predicted as below.

$$\Phi_{ReSe2\_pristine} = \Phi_{tip} - 0.33\ \mathrm{V} = 4.48\ \mathrm{eV}$$

$$\Phi_{ReSe2\_HCl\text{-}doped} = \Phi_{tip} - 0.2\ \mathrm{V} = 4.61\ \mathrm{eV}$$
